# A Phenothiazine-HPQ Based Fluorescent Probe with a Large Stokes Shift for Sensing Biothiols in Living Systems

**DOI:** 10.3390/molecules26082337

**Published:** 2021-04-17

**Authors:** Yan Zheng, Peng Hou, Yu Li, Jingwen Sun, Hongxia Cui, Haiyan Zhang, Song Chen

**Affiliations:** 1 Scientific Research Department, Qiqihar Medical University, Qiqihar 161006, China; wo1988jiao19@qmu.edu.cn (Y.Z.); hai1685@sina.com (H.Z.); 2 College of Pharmacy, Qiqihar Medical University, Qiqihar 161006, China; houpeng1982@163.com (P.H.); li18445286985@163.com (Y.L.); sewage2010@163.com (J.S.); xutianfang@sohu.com (H.C.)

**Keywords:** MCF-7 cells, fluorescent probe, biothiols, phenothiazine

## Abstract

Due to the redox properties closely related to numerous physiological and pathological processes, biothiols, including cysteine (Cys), homocysteine (Hcy) and glutathione (GSH), have received considerable attention in biological science. On account of the important physiological roles of these biothiols, it is of profound significance to develop sensitive and selective detection of biothiols to understand their biological profiles. In this work, we reported an efficient fluorescent probe, **PHPQ-SH**, for detecting biothiols in vitro and vivo, based on the phenothiazine-HPQ skeleton, with DNBS (2,4-dinitrobenzenesulfonate) as the response unit. Probe **PHPQ-SH** exhibited brilliant sensing performances toward thiols, including a large Stokes shift (138 nm), excellent sensitivity (for GSH, LOD = 18.3 nM), remarkable fluorescence enhancement (163-fold), low cytotoxicity, rapid response (8 min), and extraordinary selectivity. Finally, the probe **PHPQ-SH** illustrated herein was capable of responding and visualizing biothiols in MCF-7 cells and zebrafish.

## 1. Introduction

Biothiols including cysteine (Cys), homocysteine (Hcy), and glutathione (GSH) are vital members of amino acids, which have received considerable attention in biological science because of the redox properties closely related to numerous physiological and pathological processes [1,2,3]. For instance, cysteine (Cys), as a precursor of acetyl CoA and taurine, plays important functions in protein functionality and metabolism. Aberrant levels of Cys are correlated with poor growth, muscle and fat loss, skin lesions and lethargy [4,5]. Glutathione (GSH), as the most abundant endogenous biothiol, is essential for maintaining biological redox homeostasis by scavenging free radicals and peroxides. Data studies showed that many clinical diseases, such as human immunodeficiency disease (HIV), liver damage, leukopenia, and some cancers are directly associated with irregular levels of GSH [6,7,8]. Homocysteine (Hcy) is an important influencing factor of cardiovascular and Alzheimer’s disease. In addition, elevated levels of Hcy in plasma will lead to folate and vitamin B_12_ deficiency, nervous system defects, and osteoporosis [9,10,11]. On account of the important physiological roles of these biothiols, it is of profound significance to develop sensitive and selective detection methods for biothiols to understand their biological profiles.

In recent years, several detection techniques have been exploited for the detection of biothiols, such as high-performance liquid chromatography (HPLC), chemiluminescence method, colorimetric assays, electrochemical analysis and fluorescent detection [12,13,14,15,16]. Among them, fluorescence detection approaches, owing to their advantages of excellent sensitivity, low cost, high spatiotemporal resolution, ease of observation and noninvasive detection, have attracted interest in biological imaging analysis, clinical disease diagnosis, food detection, and environmental detection [17,18,19,20]. At present, a number of fluorescent probes have been designed for biothiols imaging [21,22,23,24,25,26,27,28,29]. Despite great efforts, some defects associated with current sensors, including low selectivity, complicated syntheses, low biocompatibility or a small Stokes shift (<100 nm), still remain. In fact, fluorophores with large Stokes shifts can vastly reduce autofluorescence and self-quenching, resulting from the minimal overlap of emission and excitation spectra, to improve detection accuracy [30,31,32]. Therefore, developing a sensitive and specific fluorescent probe with improved sensing performance for monitoring the existence of biothiols in biological systems is still in high demand.

A few fluorescent probes with an eligible Stokes shift for thiols were reported in our previous works [24,33], which exhibited outstanding characteristics such as high selectivity, sensitivity and rapid detection. As an extension of our work, by incorporating the phenothiazine moiety into the 2-(2′-hydroxyphenyl)-4(3H)- quinazolinone (HPQ), we developed a novel chromophore 2-(10-butyl-2-hydroxy-10H-phenothiazin-3-yl) quinazolin-4(3H)-one, **PHPQ**, which exhibited strong green fluorescence, simple synthesis route, good photostabilities and a remarkable Stokes shift. These prominent optical characteristics led us to conclude that **PHPQ** could serve as an ideal candidate for the construction of fluorescent sensors. Moreover, studies have proven that 2, 4-dinitrobenzenesulfonate (DNBS) is a highly selective and sensitive response site for biothiols [33]. Thus, an efficient fluorescent probe **PHPQ-SH** for detecting biothiols in vitro and vivo has been exploited, with **PHPQ** as the fluorophore linked to DNBS as the response unit. Free probe **PHPQ-SH** showed little fluorescence in the green region because of a remarkable photo-induced electron transfer (PET) effect from **PHPQ** to DNBS moiety. Once biothiols were added, the conversion of **PHPQ-SH** to **PHPQ** destroyed the PET process, giving the promising fluoresces in the green region. Importantly, by laser confocal microscope, the capability of **PHPQ-SH** for imaging biothiols in living MCF-7 cells and zebrafish models was successfully achieved. Compared with our previous works (Table S1), probe **PHPQ-SH** in this work had the following advantages: (1) A large Stokes shift (138 nm); (2) a significant turn-on fluorescence response (163-fold); (3) **PHPQ-SH** showed high sensitivity towards thiols (the detection limit was as low as 18.3 nM); and (4) probe **PHPQ-SH** was successfully applied to monitor levels of GSH in realistic samples.

## 2. Results

### 2.1. Design and Synthesis of PHPQ-SH

**Figure 1:**
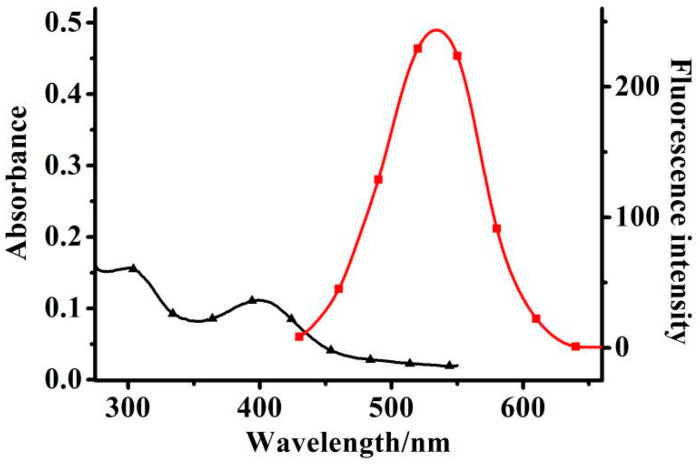
Absorption spectra (black) and emission spectra (red) of **PHPQ**.

**Scheme 1:**
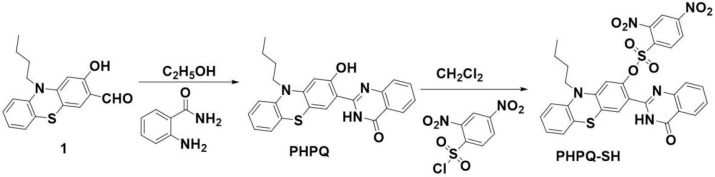
The synthesis route of **PHPQ-SH**.

**Figure 2:**
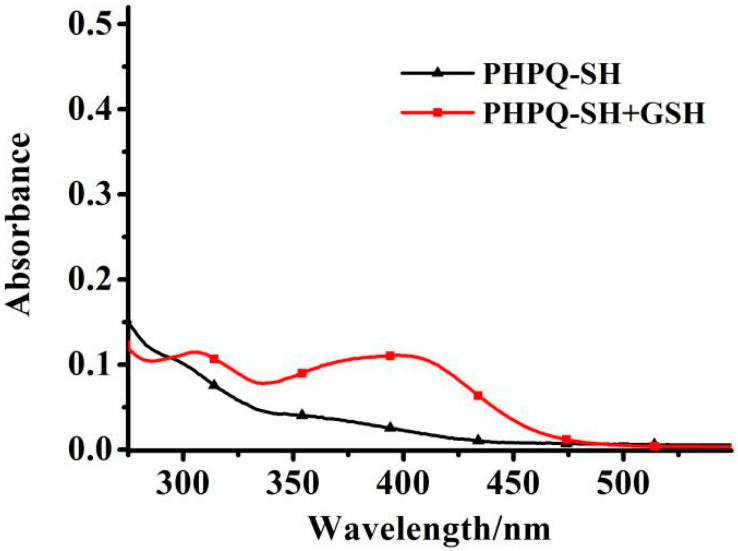
The absorption spectra of probe **PHPQ-SH** reacted with (red lines) and without GSH (Glutathione) (black lines).

**Scheme 2:**
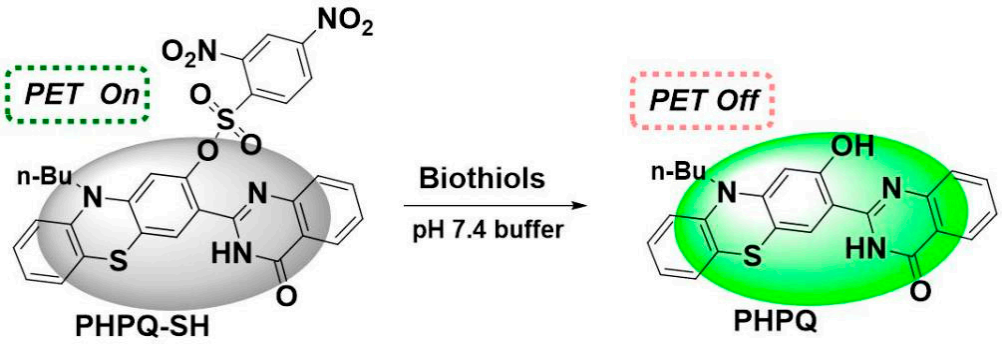
The mechanism of **PHPQ-SH** towards biothiols.

Due to its intense luminescence, good biocompatibility, large Stokes shift, and excellent photostability, 2-(2′-hydroxyphenyl)-4(3H)-quinazolinone (HPQ) has gathered significant interest [34,35]. Herein, probe **PHPQ-SH** was designed to use HPQ derivative **PHPQ** (Figure 1) as the chromophore and the DNBS group as the response site (Scheme 1). When the probe was subjected to biothiols, the masking DNBS moiety can be facilely cleaved from **PHPQ-SH** to release the highly fluorescent **PHPQ** (Figure 2), leading to the turn-on detection of biothiols (Scheme 2). On account of above considerations, a phenothiazine-HPQ based fluorescent probe **PHPQ-SH**, with a large Stokes shift for sensing biothiols, was synthesized. The chemical structures of **PHPQ** and **PHPQ-SH** were characterized by ^1^H NMR, HRMS and ^13^C NMR (Figures S7–S12).

### 2.2. Spectral Response of Probe PHPQ-SH towards Biothiols

**Figure 3:**
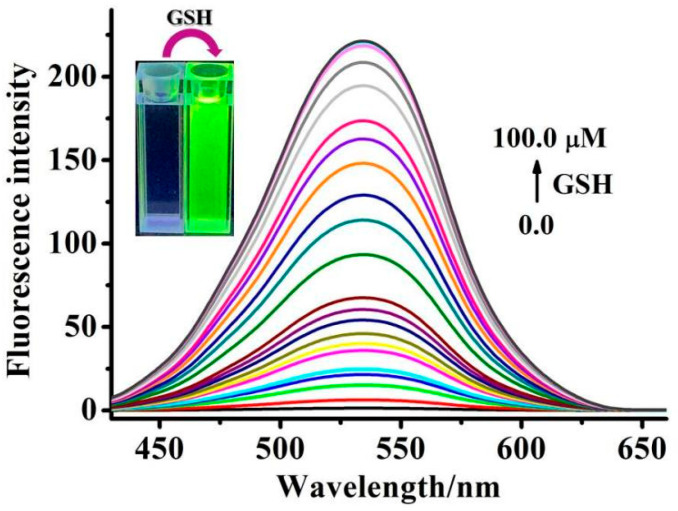
Fluorescent spectral changes of **PHPQ-SH** (10.0 µM) with different concentrations of GSH (0.0–100.00 µM) (insert: the color of fluorescent responses of **PHPQ-SH** towards GSH).

**Figure 4:**
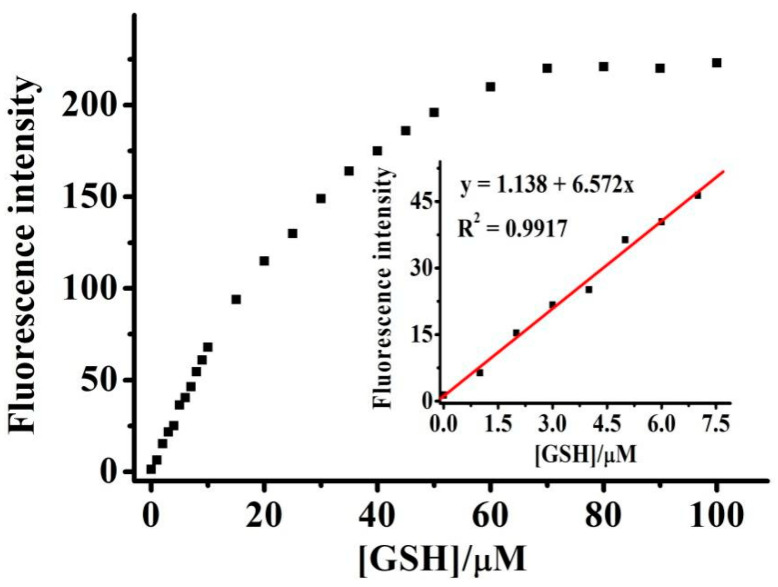
The relationship between fluorescence intensities at 535 nm of **PHPQ-SH** (10.0 µM) and GSH concentrations (insert: linear fit of fluorescence intensities in the range of 0.0–7.0 µM GSH concentration).

First, we investigated the sensing properties of **PHPQ-SH** toward thiols in a PBS/CH_3_CN solution (*v*/*v* = 1/1, pH 7.4). As seen in Figure 3, the free **PHPQ-SH** (10.0 µM) displayed an extremely weak fluorescence (Φ = 0.01). After adding GSH (0.0–100.0 µM), a green-colored fluorescence emission peak at 535 nm gradually increased. About a 163-fold enhancement in the emission intensity was obtained in the presence of 10.0 equiv. of GSH. Notably, the Stokes shift for **PHPQ-SH** (10.0 µM) in response to GSH (100.0 µM) was as high as 138 nm, which was positive for bioimaging in living systems. The large fluorescence changes in spectral properties in **PHPQ-SH** solution after the addition of GSH indicated that **PHPQ** (Φ = 0.22) was produced. The strategy in this work was also verified by the HRMS spectra (Figure S13). The mixture of **PHPQ-SH** with GSH (*m*/*z* = 416.1421) and **PHPQ** (cal. 416.1433) nearly had a same molecular weight. The excellent linear response in Figure 4 indicated that the emission intensity at 535 nm increased linearly with the concentration of GSH (y = 1.138 + 6.572x, R^2^ = 0.9917), increasing from 0.0 to 7.0 μM (for Cys, y = 3.721 + 7.334x, R^2^ = 0.9900) (for Hcy, y = −0.195 + 4.739x, R^2^ = 0.9931) (Figures S1–S4). The detection limit of probe **PHPQ-SH** for GSH was further calculated to be 18.3 nM (for Cys, LOD = 20.1 nM) (for Hcy, LOD = 20.6 nM), based on the LOD = 3 σ/s. Probe **PHPQ-SH** demonstrated high sensitivity for the identification of biothiols, with a large Stokes shift in a turn-on mode.

### 2.3. Selectivity and Interference Studies of PHPQ-SH

**Figure 5:**
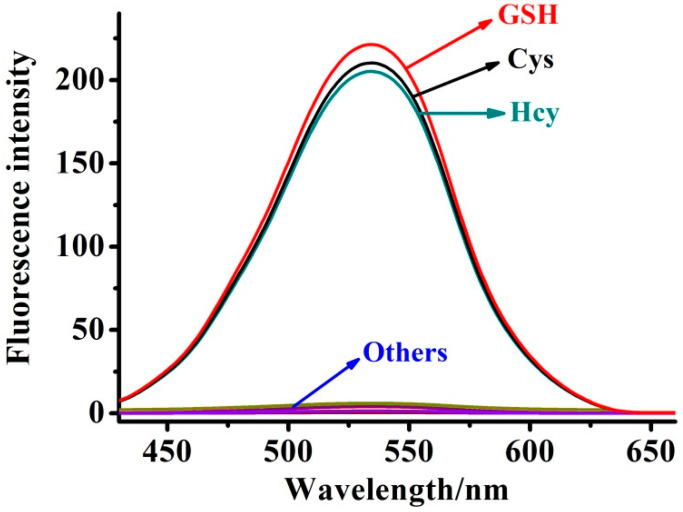
Relative fluorescence intensities at 535 nm of **PHPQ-SH** (10.0 µM), with the addition of various species (100.0 µM for Ala, Arg, Glu, Asp, Ser, Lys, Thr, Val, Tyr, Pro, Trp, Leu, Phe, Gly, Ile, Met, His, Gln, Asn, GSH, Cys, Hcy).

**Figure 6:**
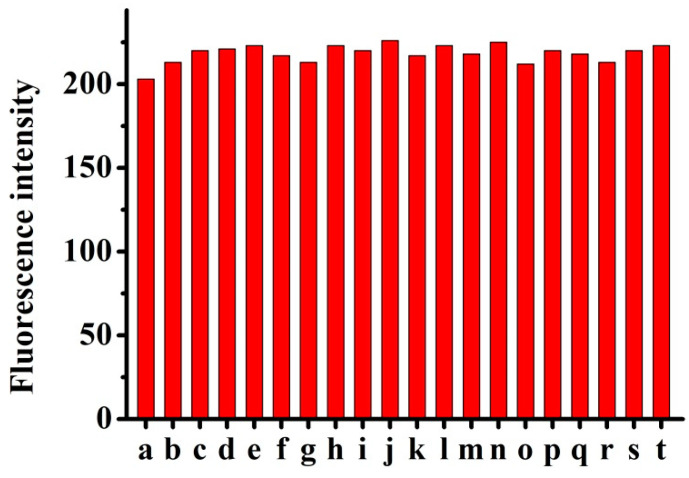
Fluorescence response of **PHPQ-SH** (10.0 µM) towards GSH (100.0 µM) with the competition analytes (a–t: 100.0 µM for Ala, Arg, Glu, Asp, Ser, Lys, Thr, Val, Tyr, Pro, Trp, Leu, Phe, Gly, Ile, Met, His, Gln, Asn, GSH).

To evaluate the availability of a fluorescent probe selective towards biothiols, the reactivity of **PHPQ-SH** towards various amino acids was studied. As displayed in Figure 5, upon the addition of representative amino acids (including Ala, Arg, Glu, Asp, Ser, Lys, Thr, Val, Tyr, Pro, Trp, Leu, Phe, Gly, Ile, Met, His, Gln, Asn), the fluorescence intensities of the testing solution (100.0 µM) were barely varied compared with that of the initial probe **PHPQ-SH** (10.0 µM). On the contrary, a prominent enhancement of the fluorescence intensity was only triggered in the case of biothiols (GSH, Cys, Hcy). Furthermore, the performance of **PHPQ-SH** (10.0 µM) for recognizing GSH in the presence of (100.0 µM) competitive amino acids (Ala, Arg, Glu, Asp, Ser, Lys, Thr, Val, Tyr, Pro, Trp, Leu, Phe, Gly, Ile, Met, His, Gln, Asn, GSH) was investigated (Figure 6). As expected, no significant interference of **PHPQ-SH** in the detection of GSH with other coexisted amino acids was observed. As shown in Figure S5, the presence of relevant ions (100.0 μM for Cl^−^, NO_3_^−^, SO_4_^2−^, PO_3_^4−^, Ca^2+^, Cu^2+^, Na^+^) also caused no effect on the fluorescence intensity of **PHPQ-SH**. The result indicated that **PHPQ-SH** can be served as a specific indicator for biothiols rather than other amino acids.

### 2.4. Effects of Response Time and pH

**Figure 7:**
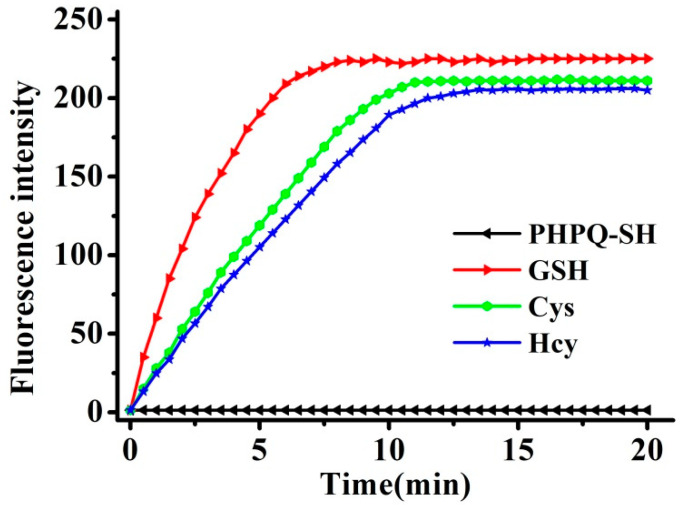
Time-dependent fluorescence intensities at 535 nm of **PHPQ-SH** (10.0 µM) with and without biothiols (100.0 µM).

**Figure 8:**
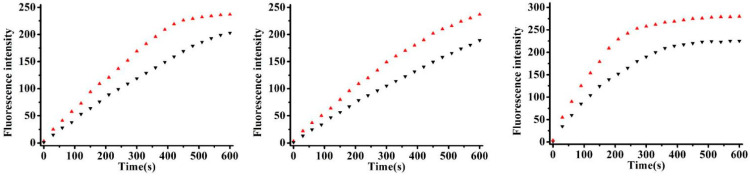
Time-dependent fluorescence intensities at 535 nm of **PHPQ-SH** (10.0 µM) with different thiols (from left to right: Cys, Hcy, GSH) at different temperature (black▼: 25 °C; red▲:37 °C).

The kinetic spectra analysis of probe **PHPQ-SH** upon introducing biothiols (GSH, Cys and Hcy) was monitored at 25/37 °C temperature (Figure 7). The time-dependent fluorescence intensity of **PHPQ-SH** (10.0 µM) at 535 nm indicated that the probe itself had high photostability, with no fluorescence change at test temperature. However, the fluorescence intensities can increase rapidly at the beginning and level out within 8 min after adding GSH (100.0 µM) to the solution of **PHPQ-SH** (10.0 µM) at 25 °C. Moreover, a similar quick fluorescence intensity enchantment appeared with probe **PHPQ-SH** in response to Cys and Hcy at 25 °C. It is obvious that probe **PHPQ-SH** could be used as a real-time candidate for biothiols’ determination. In addition, the reaction time of probe **PHPQ-SH** with thiols at 37 °C was investigated. The results are shown in Figure 8, indicating that the reaction was accelerated compared with the reaction at room temperature. The observed rated constants at 37 °C were found to be 4.458, 4.124 and 7.312 min^−1^ for Cys, Hcy and GSH, respectively.

**Figure 9:**
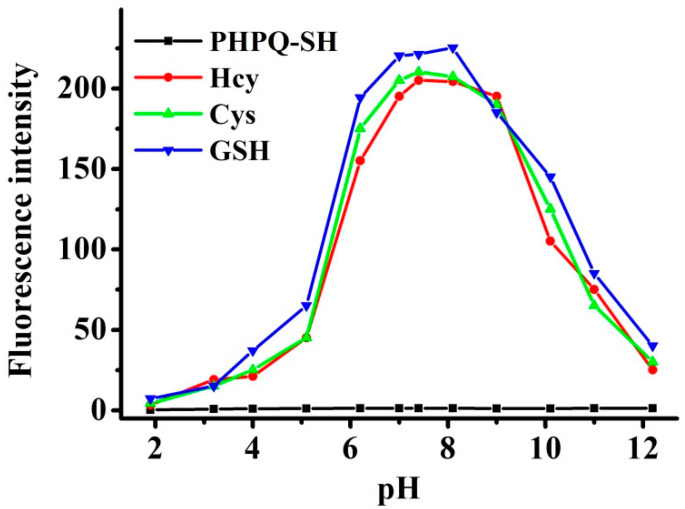
The pH response curve of **PHPQ-SH** (10.0 µM) towards biothiols (100.0 µM) at fluorescence intensities.

In order to assess the possibility of practical use for probe **PHPQ-SH** in biological systems, the effect of pH is considered an essential factor. The behavior of probe **PHPQ-SH** at various pH values was conducted through recording the fluorescence spectra. As shown in Figure 9, in the absence of GSH, an almost horizontal fluctuation curve of fluorescence intensity was observed for free **PHPQ-SH** (10.0 µM) over a broad pH range from 2.0 to 12.0, which demonstrated a strong stability of **PHPQ-SH** with pH. After adding biothiols (GSH, Cys, Hcy, respectively) (100.0 µM), remarkable fluorescence signal enhancements of systems were seen in the pH range of 6.0–9.0. The optimal working range suggested that probe **PHPQ-SH** could be used to detect biothiols in biological systems. Meanwhile, the changes of fluorescence intensity in different concentrations of buffer solution were studied (Figure S6). They indicated that the fluorescence intensity of the reaction system increased slightly with the increase of buffer concentration, which had a negligible impact on the fluorescence response of the test system.

### 2.5. Cell Imaging

**Figure 10:**
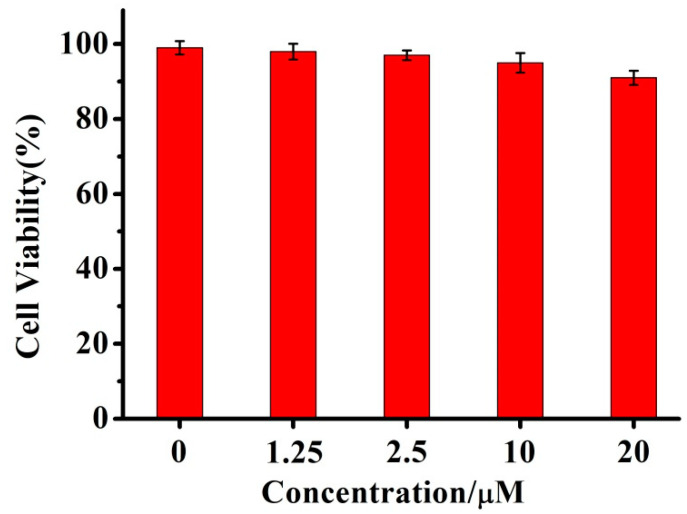
Cell viability of **PHPQ-SH** via the standard MTT assay of MCF-7.

**Figure 11:**
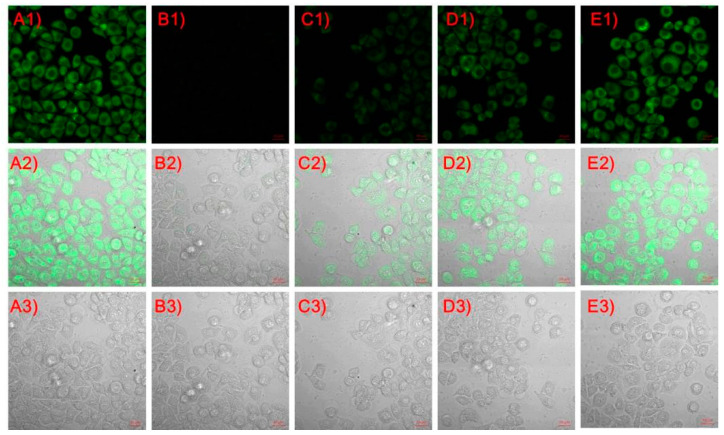
Confocal microscopy images in MCF-7 cells with **PHPQ-SH** (10.0 µM). (**A1**–**A3**): adding 10.0 µM **PHPQ-SH**; (**B1**–**B3**), (**C1**–**C3**), (**D1**–**D3**), (**E1**–**E3**): NEM loaded, 10.0 µM of **PHPQ-SH**-treated cells for 30 min, then adding 0.0 µM, 15.0 µM, 50.0 µM, and 100.0 µM of GSH, respectively.

Inspired by the aforementioned excellent fluorescent characteristics of **PHPQ-SH**, cell imaging performance for biothiols determination was investigated with MCF-7. As presented in Figure 10, standard MTT assays showed that cell viability was estimated to be as high as 91% after 24 h of incubation at different concentrations of 0.0–20.0 μM of **PHPQ-SH**, which confirmed that **PHPQ-SH** had low cytotoxicity and was suitable for intracellular biothiols detection. Next, fluorescence imaging experiments were carried out to evaluate the capability of sensing biothiols in living cells. When the cells were stained with 10.0 μM **PHPQ-SH** alone, a strong green fluorescence was captured under the confocal microscope (Figure 11A1,A2). In contrast, the cells were preincubated with NEM (as thiol scavenger, 1.0 mM) and treated with probe **PHPQ-SH** (10.0 µM); no fluorescence output in the cells was observed (Figure 11B1, B2). Moreover, after further treatment of the cells with increasing concentrations of GSH (15.0 µM, 50.0 µM, 100.0 µM), fluorescence intensity enhanced significantly (Figure 11C1,D1,E1), indicating that **PHPQ-SH** has the potential to be applied in the quantification of biothiols with dose dependently intensified.

### 2.6. Imaging Biothiols in Zebrafish

**Figure 12:**
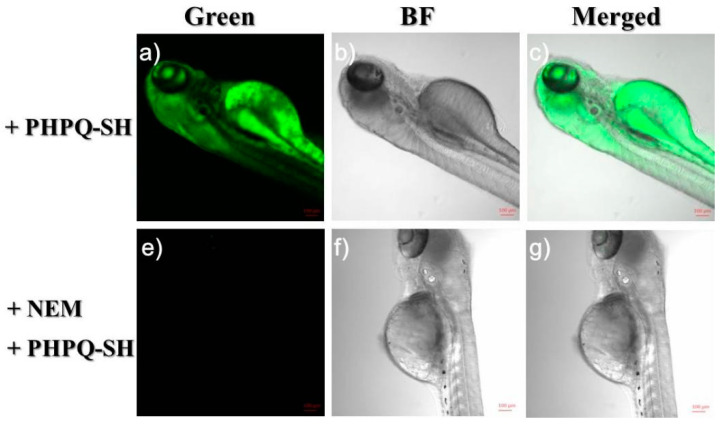
Confocal images in zebrafish with **PHPQ-SH** (10.0 µM). (**a**–**c**): **PHPQ-SH** (10.0 µM). (**e**–**g**): NEM (1.0 mM), then adding **PHPQ-SH** (10.0 µM).

Based on the cell imaging performance for biothiols determination, the capability of **PHPQ-SH** to visualize biothiols in zebrafish was carried out using a laser confocal microscope. As shown in Figure 12, when zebrafish were incubated with **PHPQ-SH** (10.0 μM) for 30 min, an intense green fluorescence appeared (Figure 12a,c), suggesting that endogenous biothiols could be conveniently detected with **PHPQ-SH**. In a control experiment, zebrafish were pretreated with NEM (1.0 mM) for 30 min, and then incubated with **PHPQ-SH** (10.0 μM) for another 30 min; negligible fluorescence (Figure 12e,g) in green channel was obtained. These data clearly implied that **PHPQ-SH** could successfully detect biothiols in zebrafish, with a brilliant performance.

### 2.7. Detection of GSH in Real Sample

Finally, the practical application of the probe **PHPQ-SH** was evaluated by measuring GSH in spiked urine sample. The recoveries and the relative errors of the proposed methods are listed in Table S2. Probe **PHPQ-SH** exhibited a recovery range (98.6%–101.4%) for GSH detection in samples, and the relative standard deviations (RSD) were all less than 2.13%. The results indicated that the probe **PHPQ-SH**, as a fluorescent sensor, could be used for the detection of GSH with good recovery and precision.

## 3. Materials and Methods

### 3.1. Materials and Instruments

All reagents were commercially available and were not further depurated for use. The UV-Vis spectra and emission spectra were obtained on a UV-2450 (Shimadzu, Tokyo, Japan) spectrophotometer and a RF5301PC (Shimadzu, Tokyo, Japan) spectrophotometer, slit: 5/5 nm. ^1^H NMR and ^13^C NMR spectra, mass spectrometric experiments, confocal fluorescence imaging and pH measurements were output from Bruker Avance 600 MHz spectrometer (Karlsruhe, Germany), Waters ^®^ Xevo G2-S QTof™ mass spectrometer (Shanghai, China), and laser scanning confocal microscope (Zeiss LSM710, Wetzlar, German), PHS-3C pH meter (Shanghai, China), respectively. 

### 3.2. Spectrum Analysis

The stock solution of **PHPQ-SH** (1.0 mM) was the prepared in CH_3_CN. Amino acid solutions (including Ala, Arg, Glu, Asp, Ser, Lys, Thr, Val, Tyr, Pro, Trp, Leu, Phe, Gly, Ile, Met, His, Gln, Asn, GSH, Cys, Hcy) for measurement were each prepared in twice-distilled water (10.0 mM). Then, the test solution was prepared by placing **PHPQ-SH** stock solution and appropriate testing analyte in phosphate buffered saline (PBS) buffer solution (pH = 7.4, containing 50% acetonitrile). The resulting mixtures were incubated well for 8 min at room-temperature and recorded by spectral measurements. The parameters of fluorescence spectra were set to λ_ex_/_em_ = 397/535 nm.

### 3.3. Cell Cytotoxicity Assay and Fluorescence Imaging

The fluorescence imaging tests to biothiols were performed in MCF-7 cells. The cells were cultured in Dulbecco’s modified Eagle’s medium (DMEM) at 5% CO_2_ and 37 °C for 24 h until the cells were overgrown. For the toxicity of **PHPQ-SH**, the cells were separately incubated with various concentrations (0.0–20.0 μM) of **PHPQ-SH** for 24 h in a 96-well plate, and then cell viability of the treated cells was measured by the MTT staining method. For confocal imaging, the MCF-7 cells were loaded on a confocal dish. The experiments were divided into three groups. The first group of MCF-7 cells was stained with **PHPQ-SH** (10.0 μM) for 30 min and photographed after washing the excess **PHPQ-SH** with PBS. Compared with the first group, the second group of MCF-7 cells was preincubated with NEM (1.0 mM) for 30 min and treated with **PHPQ-SH** (10 μM) for 30 min. In the third group, the MCF-7 cells were pretreated with NEM (1.0 mM) for 30 min, followed by incubation with **PHPQ-SH** (10.0 μM) for 30 min, and, after being placed in GSH at concentrations of 15.0, 50.0, 100 μM respectively, for another 30 min, the fluorescence imaging was performed.

### 3.4. Zebrafish Imaging

The 4-day-old zebrafish were fed in E3 embryo culture water to conduct imaging study. For zebrafish imaging experiment, zebrafish were stained with **PHPQ-SH** (10 μM) for 30 min. For the control case, zebrafish were pretreated with NEM (1.0 mM) for 30 min, followed by treatment with **PHPQ-SH** (10 μM) for another 30 min. Each fluorescence image of zebrafish was performed with confocal microscope after adopting E3 media to wash away the excess incubate reagents.

### 3.5. Sample Determination

The urine sample was treated with N-ethyl maleimide (NEM) to block the thiol groups for 1.5 h, then was diluted (1:10, *v*/*v*) with Tris-HAc buffer. For the analysis, each stock solution was added with probe **PHPQ-SH** and a known concentration of GSH (ranged from 1.0 to 20.0 µM). The resulting solution was shaken well, and then the fluorescence spectra were recorded.

### 3.6. Synthesis of **PHPQ**

2-Aminobenzamide 54.5 mg (0.4 mmol), compound 1 [36] (119 mg, 0.4 mmol) and 10 mL of anhydrous ethanol were added to a 100 mL round bottom single neck round bottom flask under argon atmosphere. The resulting mixture was stirred at refluxing temperature for 50 min. Then, 5 mg of p-toluenesulfonic acid was added and the resulting mixture was stirred at refluxing temperature for 2 h. After cooling to the room temperature, 90.8 mg of DDQ was added and the mixture was stirred at room temperature for 2 h. Finally, the reaction solution was filtered and washed with anhydrous ethanol. Dried and purified by column chromatography (mixtures of dichloromethane and ethyl acetate as eluent; 30:1, *v*/*v*) to obtain the probe **PHPQ** (53.8%). ^1^H NMR (600 MHz, DMSO) δ 14.28 (s, 1H), 12.31 (s, 1H), 8.12 (d, J = 7.2 Hz, 1H), 8.06 (s, 1H), 7.87–7.77 (m, 1H), 7.70 (d, J = 8.1 Hz, 1H), 7.50 (t, J = 7.4 Hz, 1H), 7.26–7.13 (m, 2H), 7.08 (d, J = 8.1 Hz, 1H), 6.99 (t, J = 7.4 Hz, 1H), 6.59 (s, 1H), 3.92 (t, J = 7.0 Hz, 2H), 1.79–1.63 (m, 2H), 1.42 (dd, J = 14.9, 7.4 Hz, 2H), 0.90 (t, J = 7.4 Hz, 3H). ^13^C NMR (150 MHz, DMSO) δ 161.86, 153.38, 150.07, 146.51, 143.33, 135.53, 128.11, 127.61, 126.95, 126.53, 126.02, 125.55, 123.74, 123.57, 120.84, 116.85, 113.00, 107.77, 104.75, 46.93, 28.48, 19.80, 14.10. HRMS (EI) *m*/*z* calcd for [C_24_H_21_N_3_O_2_S+H]^+^: 416.1433, Found: 416.1422.

### 3.7. Synthesis of Probe **PHPQ-SH**

Under argon atmosphere, **PHPQ** 21.0 mg (0.05 mmol), 2, 4-dinitrobenzenesulfonyl chloride 17 mg (0.065 mmol) and 30 µL triethyl amine were dissolved in 6 mL CH_2_Cl_2_. Then, the resulting mixture was stirred for 4 h at room temperature. Finally, the solvent was evaporated under reduced pressure and crude product was purified with silica gel chromatography (dichloromethane and ethyl acetate as eluent; 40:1, *v*/*v*) to afford probe **PHPQ-SH** (88.6%). ^1^H NMR (600 MHz, DMSO) δ 12.40 (s, 1H), 8.62 (d, J = 2.2 Hz, 1H), 8.22 (dd, J = 8.7, 2.2 Hz, 1H), 7.99 (dd, J = 13.8, 8.3 Hz, 2H), 7.74–7.63 (m, 1H), 7.48–7.40 (m, 2H), 7.36 (d, J = 8.1 Hz, 1H), 7.30–7.23 (m, 1H), 7.23–7.16 (m, 1H), 7.13 (d, J = 8.2 Hz, 1H), 7.03 (dd, J = 14.3, 6.8 Hz, 2H), 3.92 (t, J = 6.8 Hz, 2H), 1.67 (s, 2H), 1.41 (d, J = 7.4 Hz, 2H), 0.90 (s, 3H). ^13^C NMR (150 MHz, DMSO) δ 160.97, 150.28, 149.28, 148.00, 147.44, 145.95, 134.28, 132.94, 131.39, 128.50, 128.15, 127.33, 127.11, 126.95, 125.53, 123.59, 123.44, 122.19, 121.34, 120.59, 120.10, 116.46, 111.21, 46.50, 27.95, 19.22, 13.49. HRMS (EI) *m*/*z* calcd for [C_30_H_23_N_5_O_8_S_2_ + Na]^+^: 668.0886, Found: 668.0879.

## 4. Conclusions

In conclusion, an efficient fluorescent probe, **PHPQ-SH**, for detecting biothiols in vitro and vivo, based on the phenothiazine-HPQ skeleton, with the DNBS as the response unit, was synthesized. By taking advantage of the transformable PET process, probe **PHPQ-SH** was shown to be capable of sensitively monitoring biothiols in a turn-on signaling mode. Meanwhile, the probe **PHPQ-SH** features remarkable fluorescence enhancement (163-fold), rapid response (8 min), a large Stokes shift (138 nm), excellent sensitivity (for GSH, LOD = 18.3 nM), low cytotoxicity, and extraordinary selectivity in response to biothiols. Probe **PHPQ-SH** was also applied to monitor levels of GSH in realistic samples. Furthermore, the fluorescence imaging data clearly implied that **PHPQ-SH** could successfully detect biothiols in MCF-7 cells and zebrafish, with brilliant performances.

## Data Availability

Data is contained within the article or Supplementary Materials.

## Supplementary Materials

The following are available online. Table S1: Fluorescent probes for biothiols. Figure S1: Fluorescence response of probe PHPQ-SH (10.0 μM) upon the addition of Cys (0.0–100.0 μM) in PBS buffer. Figure S2: Fluorescence response of probe PHPQ-SH (10.0 μM) upon the addition of Hcy (0.0–100.0 μM) in PBS buffer. Figure S3: Fluorescence intensity of probe PHPQ-SH (10.0 μM) at 535 nm as a function of Cys concentration (0.0–100.0 μM) in PBS buffer. Insert: the linear relationship between fluorescence intensity and Cys at low concentrations. Figure S4: Fluorescence intensity of probe PHPQ-SH (10.0 μM) at 535 nm as a function of Hcy concentration (0.0–100.0 μM) in PBS buffer. Insert: the linear relationship between fluorescence intensity and Hcy at low concentrations. Figure S5: The selectivity at 535 nm of PHPQ-SH (10.0 μM) with the reaction of the various analytes (1–7: 100.0 μM for Cl^−^, NO^3−^, SO_4_^2−^, PO_3_^4−^, Ca^2+^, Cu^2+^, Na^+^). Figure S6: The fluorescence intensities at 535 nm of PHPQ-SH (10.0 µM) with different concentration (10.0, 20.0, 30.0, 40.0, 50.0 mM) PBS. Figure S7: 1H NMR spectrum of PHPQ in DMSO-*d*_6_. Figure S8: 13C NMR spectrum of PHPQ in DMSO-*d*_6_. Figure S9: Mass spectrum of PHPQ. Figure S10: 1H NMR spectrum of PHPQ-SH in DMSO-*d*_6_. Figure S11: 13C NMR spectrum of PHPQ-SH in DMSO-*d*_6_. Figure S12: Mass spectrum of PHPQ-SH. Figure S13: Mass spectrum of PHPQ-SH + GSH. Table S2: Determination of GSH in spiked urine sample (*n* = 5).

## Sample Availability

Samples of the compounds are not available from the authors.
