# Supplementary material for: A Phenothiazine-HPQ Based Fluorescent Probe with a Large Stokes Shift for Sensing Biothiols in Living Systems
Source: Molecules. 2021 Apr 17;26(8):2337. doi: 10.3390/molecules26082337 (PMC8072808; doi:10.3390/molecules26082337)
Supplement: Supplementary file 1 [file molecules-26-02337-s001.zip › molecules-1136297-supplementary.pdf]

Supporting information for

A phenothiazine-HPQ based fluorescent probe with a large Stokes shift for sensing biothiols in living systems

Yan Zheng [1], Peng Hou [2], Yu Li [2], Jingwen Sun [2], Hongxia Cui [2], Haiyan Zhang [1], Song Chen [b]*

[1]Scientific Research Department, Qiqihar Medical University, Qiqihar, P. R. China, 161006.

[2]College of Pharmacy, Qiqihar Medical University, Qiqihar, P. R. China, 161006.

* *Corresponding author.*

E-mail address: chensong@qmu.edu.cn

Table of contents

Page

Table S1..**S2**

Figures S1-2..**S3**

Figures S3-4..**S4**

Figures S5-6..**S5**

Figures S7-8..**S6**

Figures S9-10..**S7**

Figures S11-12..**S8**

Figures S13..**S9**

Table S2..**S10**

Table S1 Fluorescent probes for biothiols (our previous works).

Compound	λex/λem (nm)	Stokes shift (nm)	fluorescent enhancement	LOD	Response time	Reference
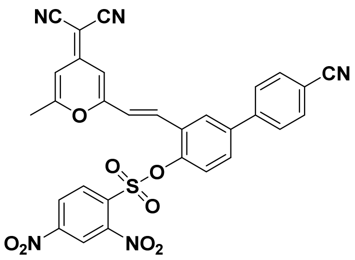	520/645	133	24-fold	21.5 nM for GSH	5 min	Spectrochim. Acta A 2020, 241, 118655
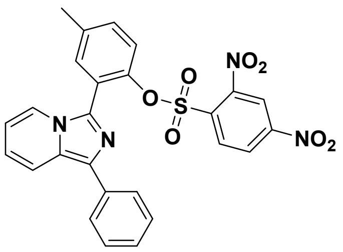	301/473	172	31-fold	20.4 nM for GSH	400s	Molecules 2019, 24, 3328
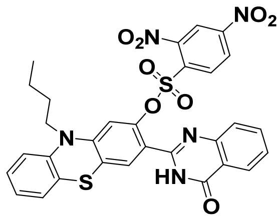	397/535	138	163-fold	18.3 nM for GSH	8 min	This work

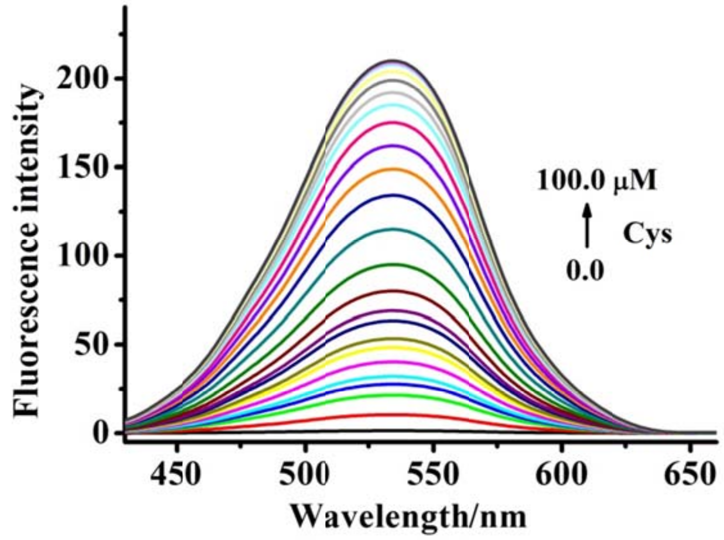

Fig. S1 Fluorescence response of probe **PHPQ-SH** (10.0 μM) upon the addition of Cys (0.0–100.0 μM) in PBS buffer.

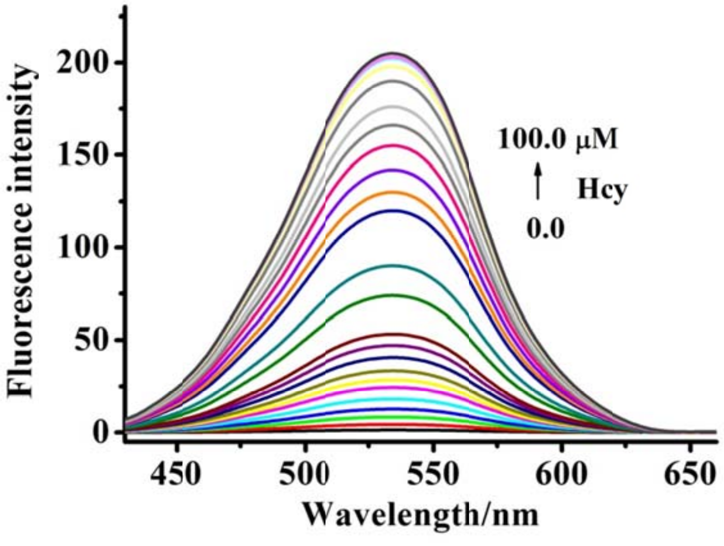

Fig. S2 Fluorescence response of probe **PHPQ-SH** (10.0 μM) upon the addition of Hcy (0.0–100.0 μM) in PBS buffer.

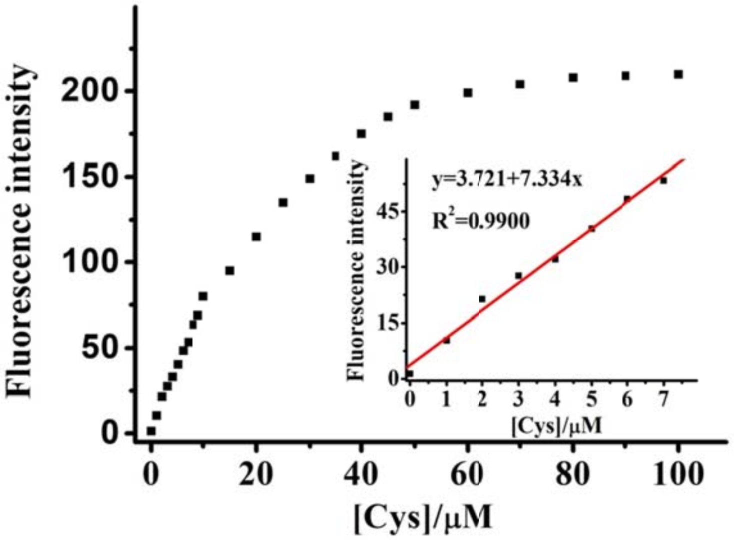

Fig. S3 Fluorescence intensity of probe **PHPQ-SH** (10.0 μM) at 535 nm as a function of Cys concentration (0.0–100.0 μM) in PBS buffer. Inset: the linear relationship between fluorescence intensity and Cys at low concentrations.

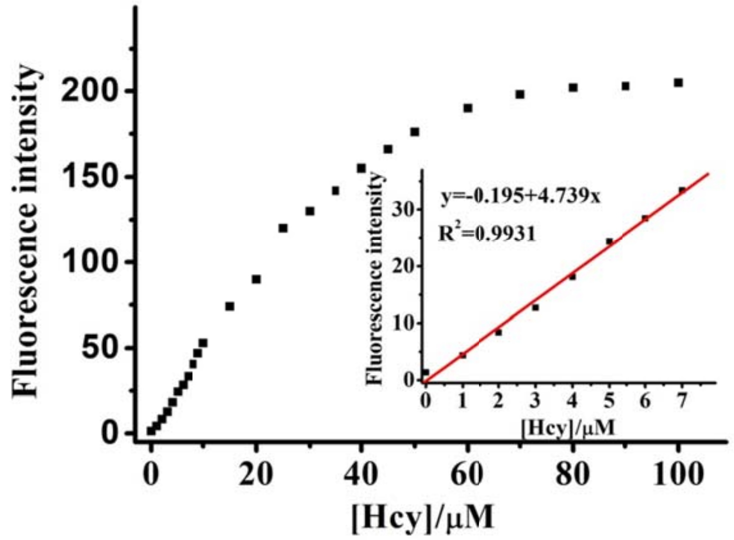

Fig. S4 Fluorescence intensity of probe **PHPQ-SH** (10.0 μM) at 535 nm as a function of Hcy concentration (0.0–100.0 μM) in PBS buffer. Inset: the linear relationship between fluorescence intensity and Hcy at low concentrations.

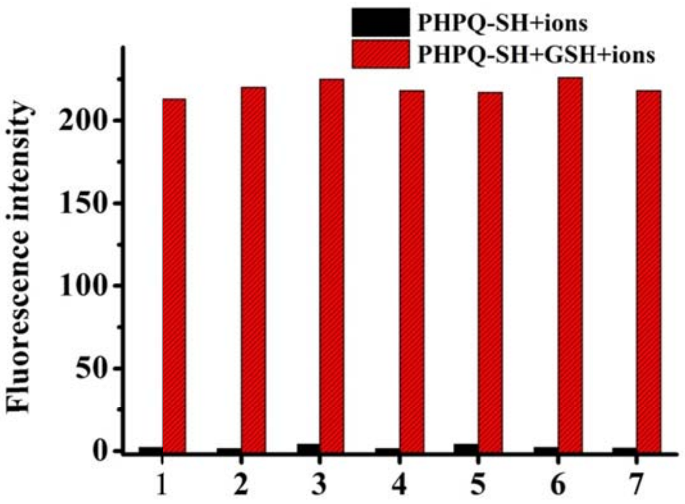

Fig. S5 The selectivity at 535 nm of **PHPQ-SH** (10.0 μM) with the reaction of the various analytes (1–7: 100.0 μM for Cl^-, NO^{3-}, SO_4^{2-}, PO_3^{4-}, Ca^{2+}, Cu^{2+}, Na^+).

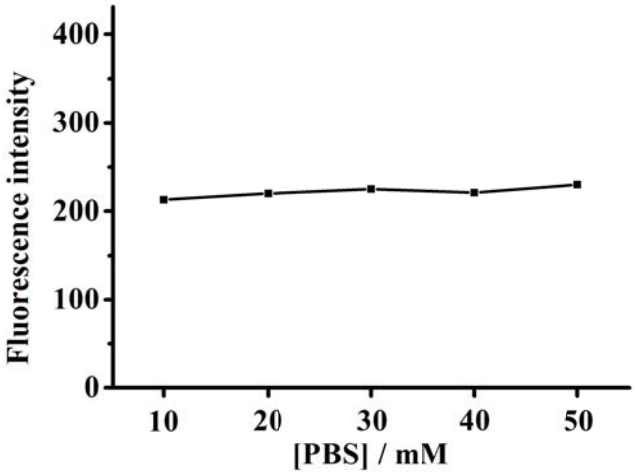

Fig. S6 The fluorescence intensities at 535 nm of **PHPQ-SH** (10.0 μM) with different concentration (10.0, 20.0, 30.0, 40.0, 50.0 mM) PBS.

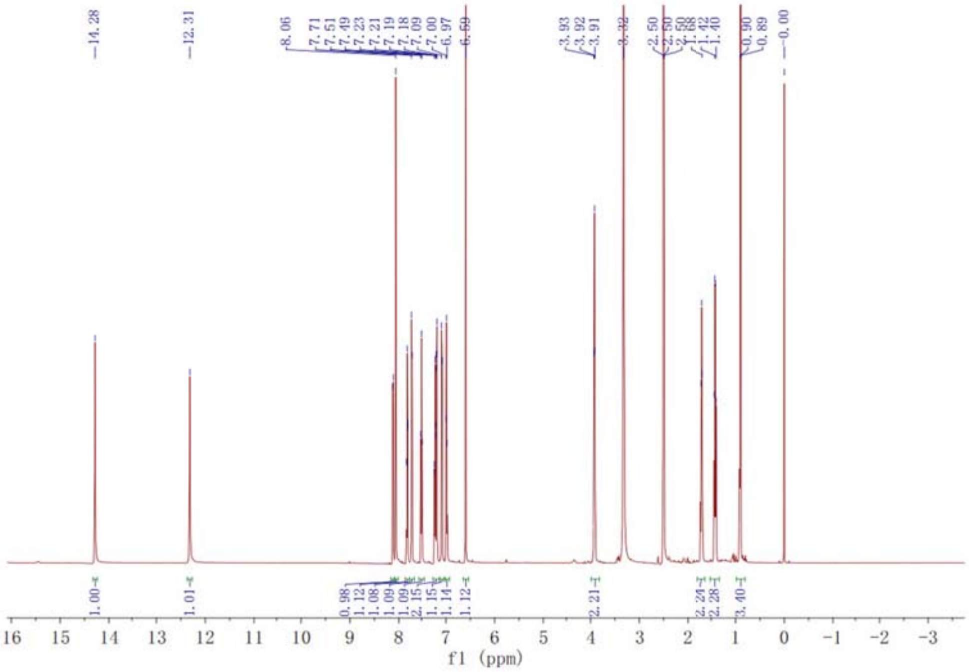

Fig. S7 ^{1}H NMR spectrum of **PHPQ** in DMSO-*d*$_6$.

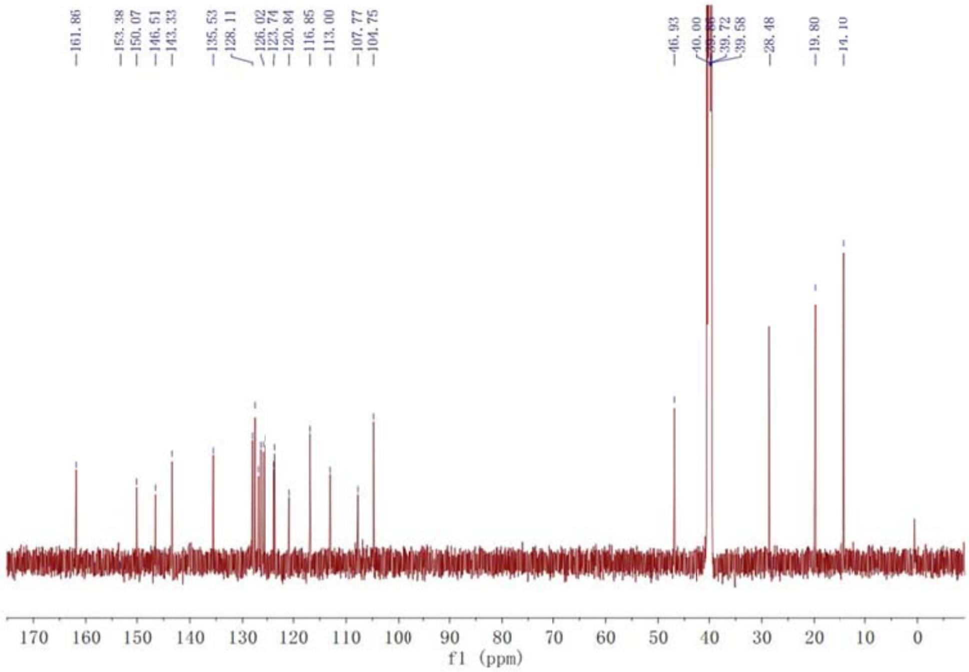

Fig. S8 ^{13}C NMR spectrum of **PHPQ** in DMSO-*d*$_6$.

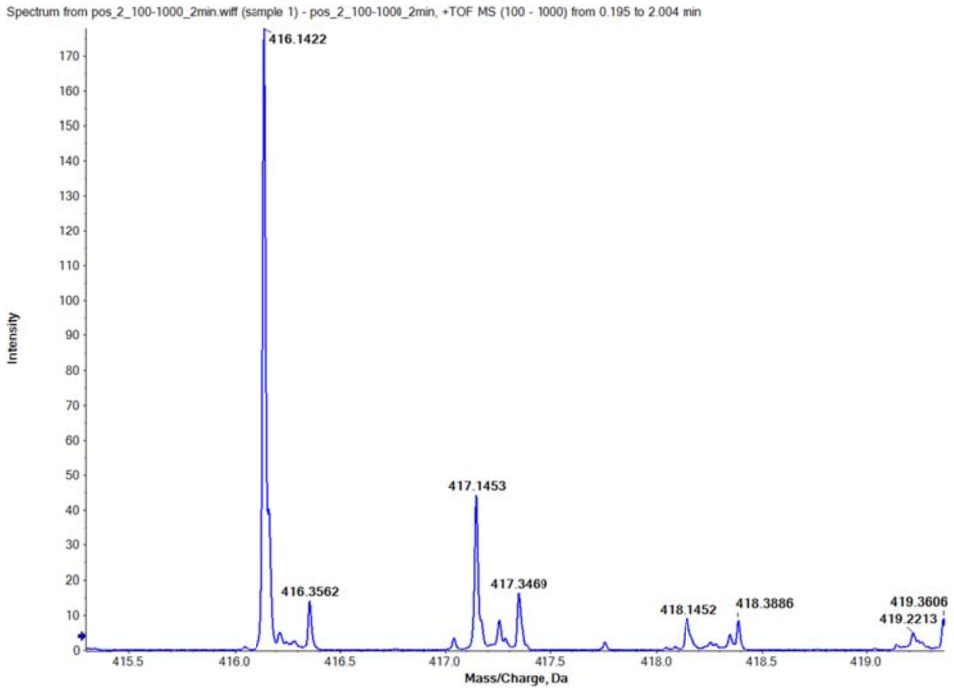

Fig. S9 Mass spectrum of **PHPQ**.

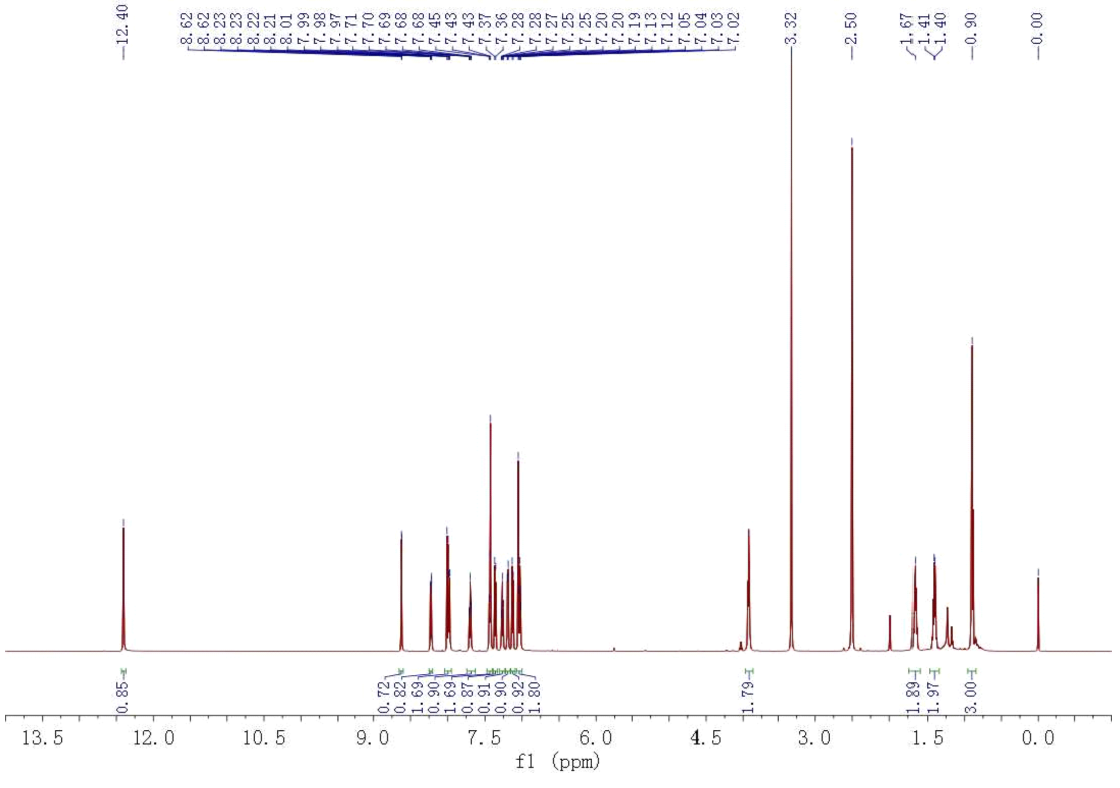

Fig. S10 ^{1}H NMR spectrum of **PHPQ-SH** in DMSO-d_6.

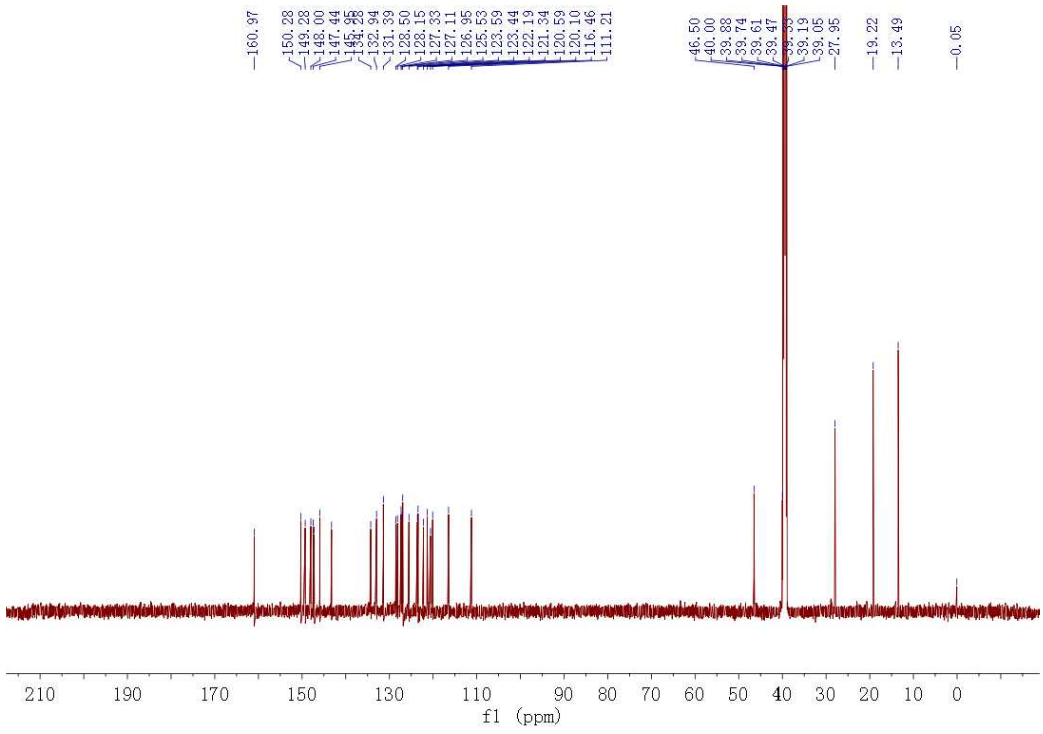

Fig. S11 ^{13}C NMR spectrum of **PHPQ-SH** in DMSO-*d*$_6$.

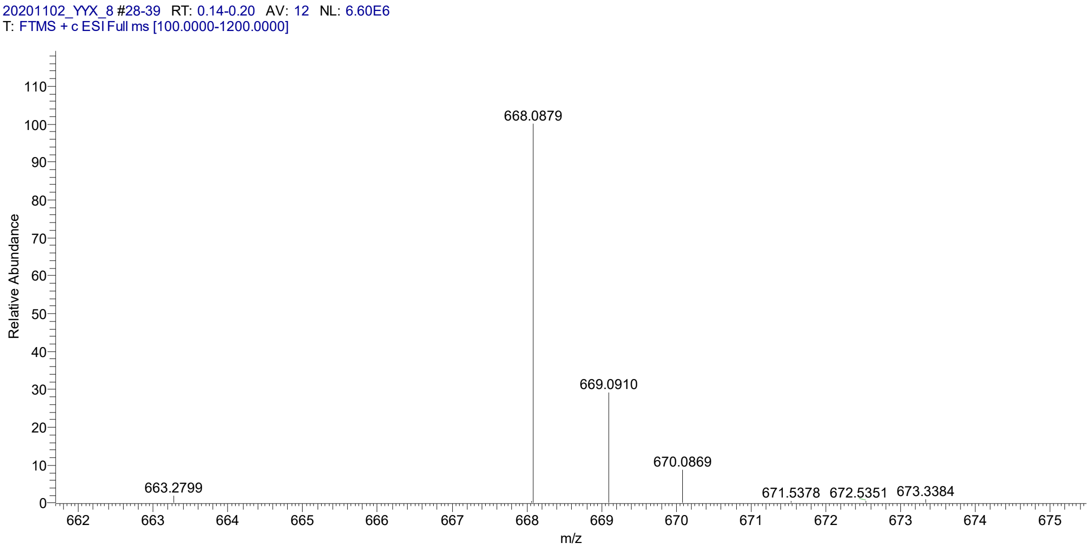

Fig. S12 Mass spectrum of **PHPQ-SH**.

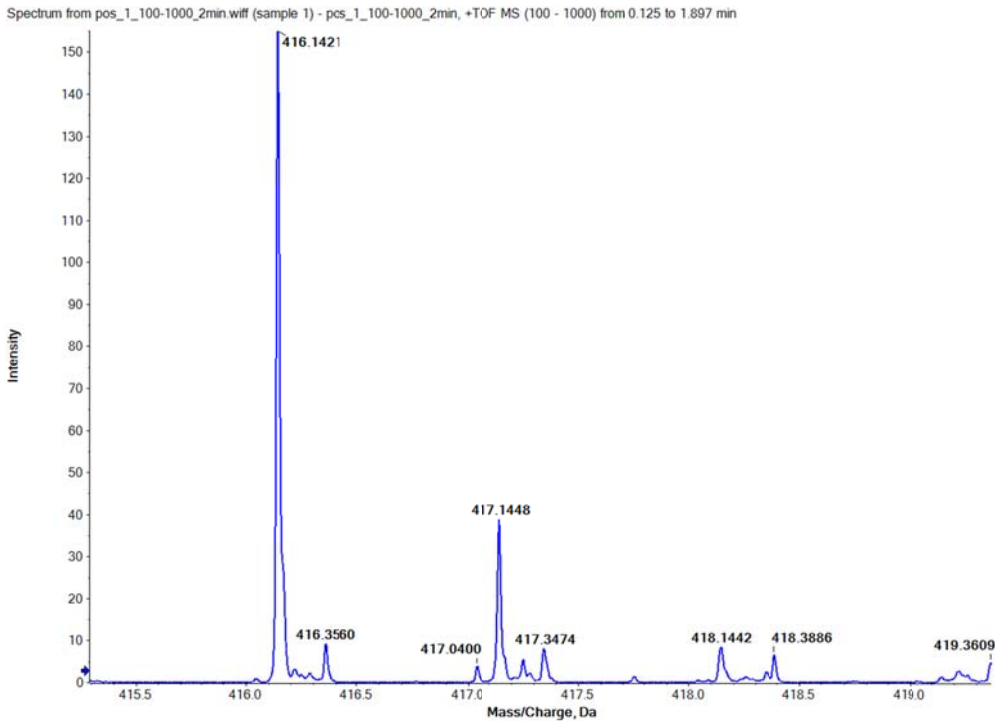

Fig. S13 Mass spectrum of **PHPQ-SH +GSH**.

Table S2 Determination of GSH in spiked urine sample (n=5)

Sample	Added (μM)	Found (μM)	Recovery(%)	Rsd(%, n=5)
1	1.0	1.014±0.034	101.4	1.47
2	5.0	5.021±0.051	100.4	2.13
3	10.0	9.863±0.012	98.6	1.64
4	20.0	20.017±0.026	100.1	2.05
